# Texas State Medical Association

**Published:** 1902-03

**Authors:** Taylor Hudson

**Affiliations:** President; Office of the President, Belton, Texas


### Texas State Medical Association.

OFFICE OF THE PRESIDENT,
BELTON, TEXAS, January 23, 1902.

DEAR DOCTOR:—I beg to advise you that the result of the vote submitted to the members of the Association was largely in favor of a change in place of next meeting, and that the city of Dallas received the largest number of votes. However, as the date of the Association meeting conflicted with that of the Confederate Veterans, I was compelled to change the date of our convention, hence the next session of the Texas State Medical Association will convene in the city of Dallas, May 6, 7, 8 and 9, 1902. It is earnestly hoped that you will make every effort to be present on that occasion, and induce your friends in the profession to attend. There are many good and useful men in Texas who have not been in the habit of attending our meetings. Let us do what we can to interest them in our work. I do not believe there has been in the past a more important session of the Association than this. Matters of vital importance must come up at this meeting and be disposed of, therefore the urgent demand for your presence. Dallas is a central point, easy of access, with abundant railroad facilities, good hotels, and is the choice of the members of the Association. The hospitality of that city is recognized all over the South. So let us unite to have one of the largest and best meetings in the history of the Association. In this connection, let me direct the attention of officers of affiliated societies to Article VII, Section 1, of the Constitution. In view of the proposed revision of the Constitution and By-Laws, it becomes the imperative duty of every affiliated society to send its delegate to the Dallas meeting.

Hoping to meet you in the metropolis of North Texas, I am,

Very sincerely yours,

TAYLOR HUDSON, M. D., President.

